# Study of coliforms and *Clostridium* bacteria inactivation in wastewaters by a pilot photolysis process and by the maturation lagoons of a low-cost nature-based WWTP

**DOI:** 10.1007/s11356-021-18184-w

**Published:** 2022-01-20

**Authors:** Juan Carlos García-Prieto, Cynthia Manuela Núñez-Núñez, José Bernardo Proal-Nájera, Manuel García-Roig

**Affiliations:** 1 Centro de Investigación y Desarrollo Tecnológico del Agua (CIDTA), Universidad de Salamanca, Campus Miguel de Unamuno, Facultad de Farmacia, Campo Charro s/n, 37080 Salamanca, Spain; 2 CIIDIR–Unidad Durango, Instituto Politécnico Nacional, Sigma 119, Fracc. 20 de Nov. II, 34220 Durango, Dgo Mexico

**Keywords:** Photolysis, Wastewater, Disinfection/inactivation, Endospores, *Clostridium*, *Coliforms*, Maturation lagoons, Constructed wetlands

## Abstract

The inactivation processes of coliform bacteria (total and fecal) and sulphito-reducing *Clostridium* bacteria (vegetative species and spores) in water maturation lagoon of a low-cost nature-based wastewater treatment plant using constructed wetlands and through processes of photolysis in a pilot photoreactor have been comparatively studied. The different inactivation mechanisms by photolysis of these bacteria have been studied following the criteria of different statistical and kinetic models. *Clostridium* disinfection treatments fit models in which two types of bacteria populations coexist, one sensitive (vegetative species) and the other (spores) resistant to the treatment, the sensitive one (94%) with an inactivation rate of *k* = 0.24 ± 0.07 min^−1^ and the resistant one (6%) with *k* = 0.11 ± 0.05 min^−1^. Total coliform photolytic disinfection also shows two populations with different physiological state. The time required to reduce the first logarithmic decimal cycle of the different types of bacteria (physiological states) are δ_1_ = 4.2 ± 0.9 and δ_2_ = 8.3 ± 1.1 min, respectively. For fecal coliform photolytic disinfection, only bacteria population, with *k* = 1.15 ± 0.19 min^−1^, is found. The results obtained confirm the photolytic disinfection processes and maturation lagoon are effective systems for *Clostridia* bacteria removal after water treatment by nature-based systems. Total removal of coliform bacteria is not achieved by maturation lagoons, but their reduction is significant using low doses of cumulative radiation.

## Introduction

The inactivation of microorganisms by ultraviolet radiation has been known for more than 100 years (Downes and Blunt 1877). The factors affecting the germicidal action of ultraviolet light (photolysis) depend on the absorption by the microorganisms of the appropriate electromagnetic energy (wavelength and irradiation power), which in turn depends on the properties of the fluid itself and the substances present in the fluid such as suspended solids, which can absorb part of this electromagnetic radiation (Lorch 1987). This electromagnetic energy affects the genetic material of the organism (DNA and/or RNA), so that the microorganisms cannot replicate and, therefore, die (Bolton and Linden 2003). An important aspect in the efficiency of this process is the existence of nucleic acid repair mechanisms called photoreactivation or photo repair (Groocock 1984; Guo et al. 2013), in which a photoreactive enzyme, after absorbing radiation, is able to repair the damage caused. This regenerative capacity occurs in bacteria and other microorganisms, but not in viruses, and its performance is related to the extent of UV damage, exposure to reactivating light, pH, and water temperature (Masschelein and Rice 2002). The photoreactivation phenomenon will require that the exposure of the microorganism to the reactivating light does not exceed 2 to 3 h after inactivation, taking into account that the degree of reactivation is a function inversely proportional to the radiation dose used.

Nowadays, nature-based wastewater treatments are an important alternative in the field of wastewater disinfection strategies to reduce pathogenic species that affect health (Vymazal 2005; Wu et al. 2016; Huang et al. 2018) and agricultural wastewater reuse (Masi and Martinuzzi 2007; Almuktar et al 2018; Nan et al. 2020). Furthermore, disinfection is required in some areas to fulfil certain directives, such as the Habitats Directive (92/43/EEC) and Bathing Water Directive (2006/7/EC). The level of disinfection of microorganism is insufficient in constructed wetlands (López et al. 2019), which is why some authors propose the use of UV photolytic reactors (Azaizeh et al. 2013; González et al. 2019) and others the use of maturation lagoons (Tanner et al. 2005; Russo et al. 2020).

Bacterial cells have always been described as an easy target for the study of disinfection and the selected microorganisms (Coliforms and *Clostridia*) to monitor the process are the major groups of bacteria covered by the regulation relating to water for human consumption (Gorchev and Ozolins 1984). On the one hand, coliform bacteria such as *Escherichia coli* are the most commonly used indicators of fecal contamination in drinking and wastewater regulations (Ashbolt et al. 2001). These Gram-negative bacteria inhabit the intestinal tract of humans and warm-blooded animals. Their presence in water is not only indicative of fecal contamination, but also of the presence of other possible enteric pathogens such as *Salmonella* spp., *Yersinia* spp., and *Shigella* spp. These enteric bacteria are responsible for minor gastrointestinal diseases (Dekker and Frank 2015).

On the other hand, the microorganisms such as spore-forming bacteria and protozoa, used as target organisms, have been shown to be much more resistant to disinfection (WHO 2011). For this reason, few articles have focused on these spore-forming bacteria, such as *Clostridium*, which have greater resistance to disinfection treatments (Dolin 1959; Ando and Tsuzuki 1986; Lanao et al. 2010). Given the extraordinary resistance of *Clostridium* spores to disinfection processes and other adverse environmental conditions, their presence in disinfected waters may indicate that the treatment has been deficient and perhaps other resistant pathogens have also survived (Payment 1999); hence the European Directive 98/83/EC proposed this species as an index of the presence of enteric protozoa and viruses in treated drinking water. Consequently, sporulating bacteria of the genus *Clostridium* have been used as indicators of the efficiency of the disinfection process (Josset et al. 2008). These bacteria are Gram-positive, anaerobic, sulphito-reducing *bacilli* that produce spores that are exceptionally resistant to adverse conditions in aquatic environments, including UV irradiation, extreme temperatures, and pH, and disinfection processes such as chlorination (Venczel et al. 1997; Dunlop et al. 2008). Like *E. coli*,* Clostridium* does not proliferate in most aquatic environments, making them very specific indicators of fecal contamination, thus being highly distributed in the environment and present in natural waters and soils. They are generally originated from human and animal fecal matters, especially present in wastewaters discharged into receiving waterways; hence they may represent a sign of remote or intermittent contamination (Talukdar et al. 2016). In addition, other authors indicated that climatic factors such as ambient temperature, rainfall, and relative humidity affect the incidence of these microorganisms. Hence, the rates of diseases could be influenced by climate change (Park et al. 2018; Oh et al. 2021).

Disinfection in constructed wetlands have different influencing factors, such as water composition, seasonal fluctuations, and local vegetation and whether fecal waste from wild birds and other animals add significantly to the total from human waste (Rahman et al. 2020). This means low-cost nature-based wastewater treatment plants cannot provide standardized performance, unlike conventional treatment plants. The pathogen removal mechanisms are also complex—most frequently including natural die-off due to starvation or predation, sedimentation and filtration, and adsorption (Alufasi et al. 2017). Therefore, it is therefore difficult to assess wetlands performance on average. In this work, the feasibility and performance of the photolysis for the inactivation of Coliforms and *Clostridia* bacteria and their spores in urban wastewaters after their treatment by low-cost nature-based wastewater treatment plants have been studied. For this, photolysis process in a pilot reactor and maturation lagoon in series with constructed wetlands has been used for this purpose.

## Materials and methods

### Photoreactor with total recirculation

**Fig. 1:**
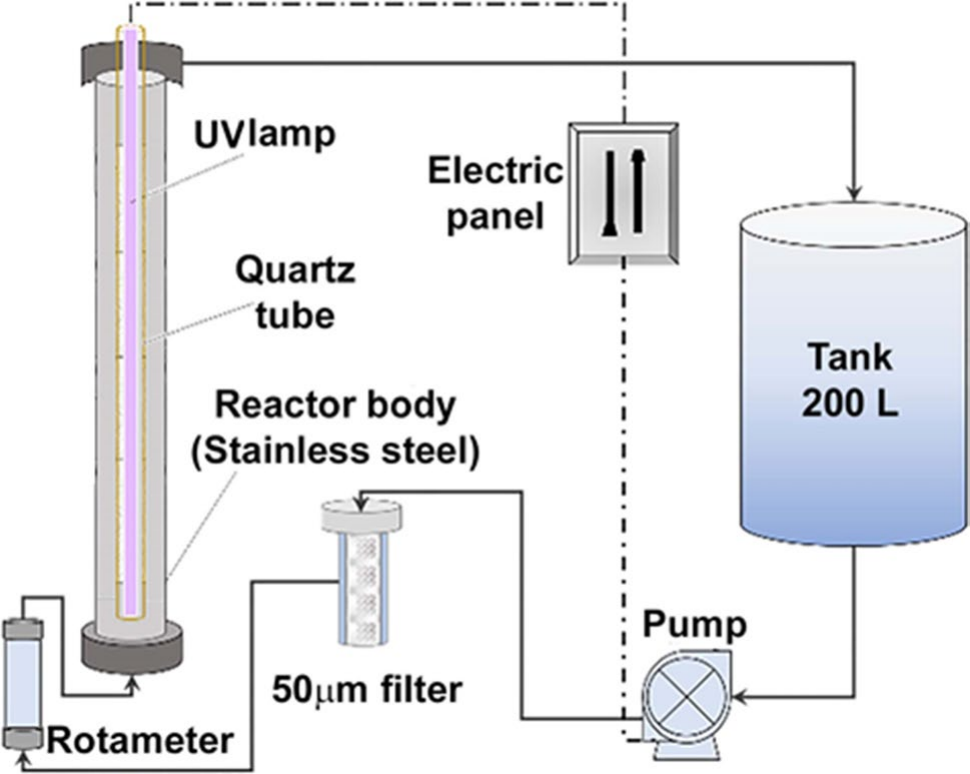
Reactor system used for photolytic inactivation processes

The photoreactor used is shown in Fig. 1. The system has a tank for the sample with a capacity of 200 L, a pump of 1 hp for recirculation of the sample in the system, and at the outlet of the pump, the water passes through a 50-μm solid filter and then through a rotameter to measure the sample flow entering the reactor body.

The reactor is characterized by maintaining a vertical piston flow with bottom inlet and upper lateral outlet; the reactor body is made of stainless steel and has a manometer on the bottom and another on the top. A 40 W Philips lamp emitting 15 W of ultraviolet radiation was used as irradiation source. The lamp was placed inside a transparent quartz tube to prevent it from entering in contact with the sample.

### Bacteria sampling, growth, sporulation, and analysis

For the study of the inactivation of Coliforms and *Clostridium* bacteria, aliquot samples were taken from the effluents of the wastewater treatment plant (WWTP) of the municipality of Monleras, belonging to the province of Salamanca, Spain, with geographical location coordinates 41° 11′ 11.99″ North, 6° 14′ 04.21″ West, was chosen as low-cost nature-based WWTP. Primary treatment is carried out in an Imhoff tank for the decantation of solids and digestion of organic matter. Secondary treatment is initially carried out in a horizontal wetland of macrophytes in flotation with other aquatic plant species, and then the water is sent to three parallel vertical subsurface flow wetlands, where the water percolates vertically through an inert substrate of sand and gravel, and finally the water reaches an artificial wetland that functions as an aerobic maturation lagoon, due to its shallow depth, for the disinfection of the water by the effect of sunlight (Arco-Alaínez 2014). This plant was designed by CIDTA’s authors, and this design was chosen by the Duero Watershed Confederation (Spain) to finance its construction within the framework of its pilot project “Singular experimental treatment of sewage discharges in small towns in the Duero river basin” (García-Prieto and Cachaza Silverio 2008).

The coliform bacteria count was carried out by the pour plate method (APHA 1995), using Chromogenic agar (Scharlau, Spain), which contains nutrients to give coloration and allows to differentiate between fecal coliforms (*E. coli*) and other coliform microorganisms present in the sample. After 24 h of incubation at 37 ℃, a visual count of the colonies was made in each plate, considering as fecal coliforms colonies those of purple coloration and as other coliforms, those that showed pink to red coloration, the total coliforms count results from the addition of fecal coliforms colonies and those of other coliforms present. As culture media for the growth and determination of *Clostridium*, iron agar and modified sulfite agar, both from SCHARLAU, were used. The culturing and counting of vegetative cells and spores of *Clostridium perfringens* are carried out following the procedure described in the Spanish Standard UNE-EN 26,461 (AENOR 2009).

### Experimental procedure

The control of the parameters involved in the determination of the inactivation efficiencies by photolysis was carried out. For each experiment, a 50 L wastewater sample from the wastewater treatment plant (Monleras) was deposited in the feed tank of the photoreactor (Fig. 1). The pump was adjusted to a recirculation flow rate of 1000 L/h; the temperature was 25 °C and in three aliquot samples, taken at each different time of the inactivation process; the colony-forming units per mL (CFU/mL) of bacteria were counted.

### Data analysis

To obtain the kinetic parameters of each model proposed for bacteria inactivation processes, it is necessary to fit the experimental kinetic data to the corresponding rate equation of the model by non-linear regression techniques. The model parameters are fitted to equations using iterative algorithms based on the least squares method. The statistical package SimFIT (Bardsley 2017) and GInaFiT (Geeraerd et al. 2005) will be used to obtain model parameters. The experimental data represent the average number of colony-forming units per mL (CFU/mL), counted at each time on triplicate samples, along the kinetic runs. To evaluate how well the model fits the experimental data obtained, in addition to the plotting of the real data along the fitting curve, two indicators are used, the coefficient of determination (*R*^2^) which provides a measure of how well observed outcomes are replicated by the inactivation model, based on the proportion of total variation of outcomes explained by the model, the closer to unity, the better the fit, and the root mean sum of squared error (MSE), which measures the average squared difference between the estimated values and the actual value, so the closer to zero, the better the goodness of fit. The kinetic parameters of bacteria inactivation in the different water samples by the photolysis processes were determined.

## Results and discussion

### Sampling tests

**Fig. 2:**
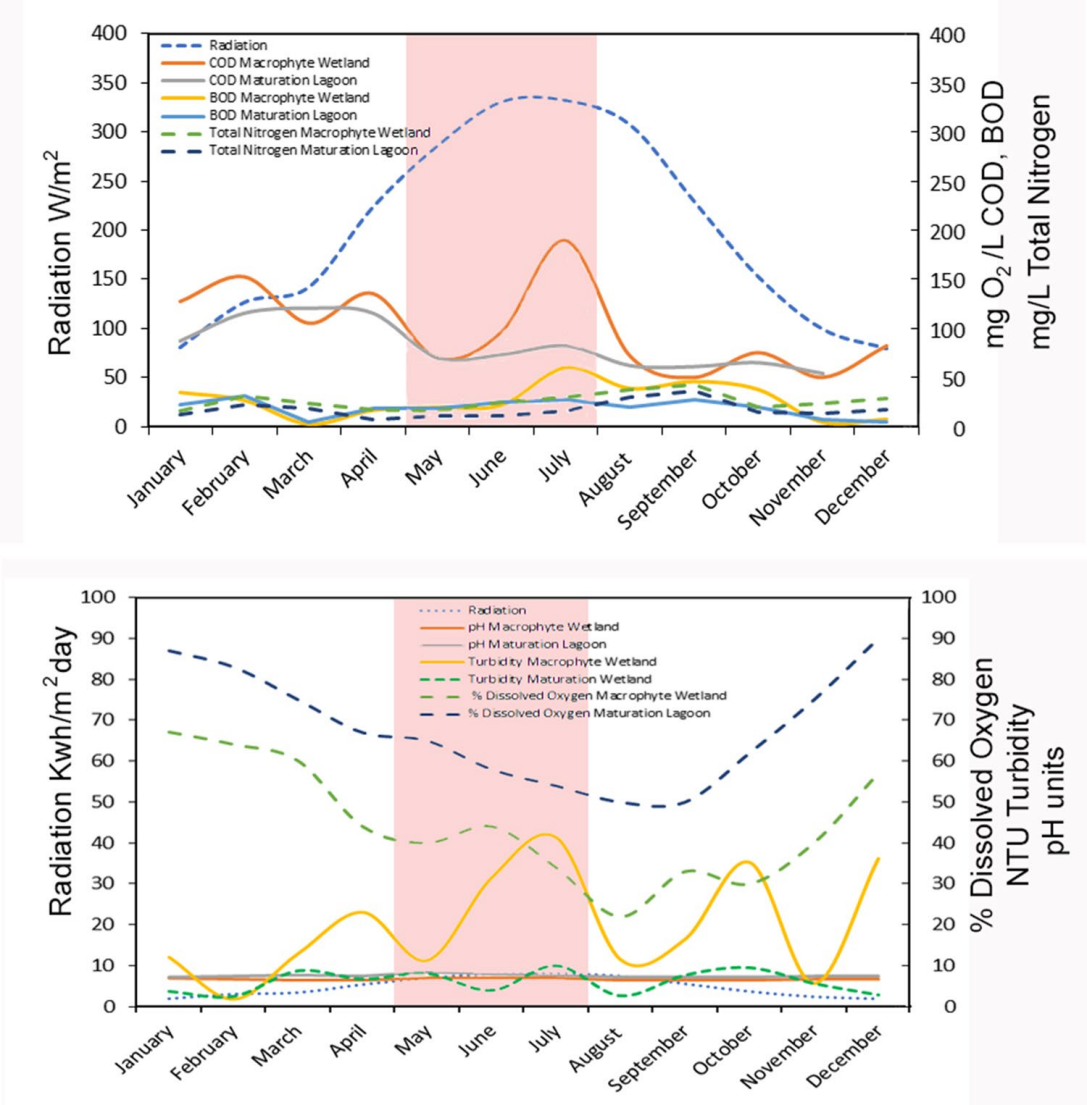
Wastewater physicochemical parameters and UV radiation characterizations at the municipal wastewater treatment plant (Monleras, Salamanca, Spain)

Several factors affect the susceptibility of microorganisms to photolytic processes. Previous studies show that it is very difficult to extrapolate the results obtained with synthetic wastewaters to real wastewater, since the growth stage of the bacteria and the synergy existing between the different microbial species present in the effluent strongly influence the results of the application of photolysis processes (Rincón and Pulgarin 2004, Rincón and Pulgarin 2007). The annual monitoring study of the main physicalchemical characteristics of Monleras WWTP wastewater samples and global UV radiation (direct and diffuse) (AEMET 2020) is shown in Fig. 2. Regarding the pH, the pH of water samples from the macrophyte wetland was 6.5–7.0, and the pH of waters from the maturation pond was between 7.23 and 7.95, throughout the year. In this sense, some researchers reported that the photochemical elimination of coliform bacteria was not affected by the pH of the solution in the range of pH 6.0–8.0 (Watts et al. 1995; Cho et al. 2004).

**Fig. 3:**
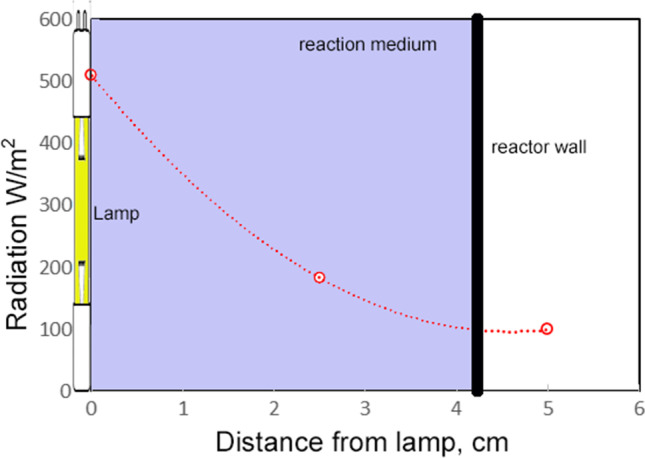
Distribution of UV lamp irradiation inside the reactor

In the case of the photolytic reactor, UV rays are predominantly emitted perpendicular to the lamp surface. To determine the radiation intensity on a surface at different distances, the intensity of the lamp at 1 m must be multiplied by the intensity factor with distance. According to the data provided by the lamp manufacturer (Koninklijke Philips), the 15 W lamp has a global UV irradiance of 144 μW/cm^2^ at a distance of 1 m. The irradiated energies at different distances within the photolytic reactor are shown in Fig. 3.

The biocidal effect of sunlight is due to the synergistic effect between radiation and temperature. Sunlight is absorbed by natural photosensitizers present in water that react with oxygen to produce highly reactive molecules such as hydrogen peroxide and superoxide ion (Khaengraeng and Reed 2005; Heaselgrave and Kilvington 2010).

Under these conditions, wastewater samples were taken in May to July (red shading Fig. 2), when optimal climatic conditions exist for the reproduction of these microorganisms and the overall UV radiation conditions are more similar to the radiation emitted by the lamp in the photolytic reactor. These bacterial inactivation processes in the tertiary wetland (maturation lagoon) of Monleras WWTP to pilot photolytic reactor were compared.

### Kinetics of bacteria inactivation processes in wastewaters treated by nature-based treatments

**Fig. 4:**
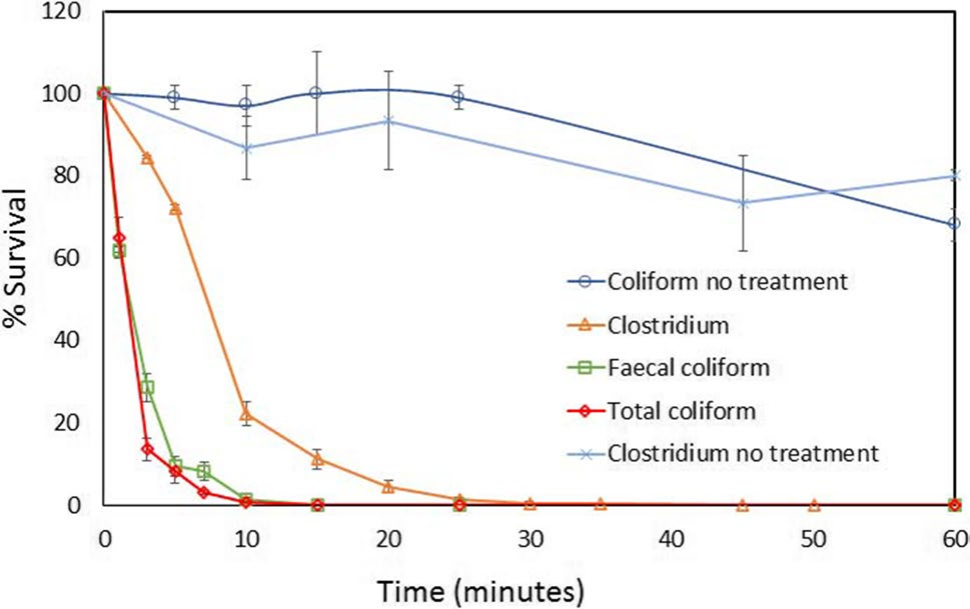
% Survival bacteria in wastewater samples from the Monleras WWTP subjected to photolysis and no light irradiation within the reactor

In the first place, the bacteria removal effect of the 50-μm filter of the reactor circuit on the bacteria present in the wastewater samples was tested. For comparative purposes, bacteria (CFU/mL) were counted in kinetic experiments carried out in the photoreactor for 60 min with macrophyte wetland of Monleras WWTP effluent samples (1) in absence of UV radiation (without lamp with filtering effect), and (b) with UV light irradiation (photolysis with filtering effect) (Fig. 4). It can be seen that the CFU/mL remained in the order of 20% in the “no treatment” experiments, which shows that the filter do not reduce the initial concentration order on their own in the reactor. It is observed how the inactivation kinetics of bacteria in the wastewater samples treated by photolysis are fast and efficient, reaching a 100% inactivation performance of the bacteria present in about 25 min of treatment.

### Kinetic modeling of photolytic processes of microbial inactivation

Different kinetic models have been proposed in the literature to explain microbial disinfection processes by photolysis under ultraviolet irradiation (Dalrymple et al. 2010). The first kinetic models described for ultraviolet disinfection are based on the Chick-Watson model (Chick 1908), which proposed a first-order model according to the following differential and integrated rate equations:

$$\mathrm r=-\frac{\mathrm{dN}}{\mathrm{dt}}=-\mathrm{kCN}$$

$$\mathrm{Ln}\frac{{\mathrm N}_{\mathrm t}}{{\mathrm N}_{\mathrm o}}=-\mathrm{kC}^{\mathrm n}\mathrm t==-\mathrm k\;\mathrm I\;\mathrm t$$

where *N* is the concentration of viable organisms (CFU/100 mL) after exposure to UV light, *N*_*o*_ is the concentration of viable organisms (CFU/100 mL) before exposure to UV light,* k* is the first-order rate constant, *C* is the concentration of disinfectant, and *n* is the number of disinfectant molecules required for microbial inactivation.

In the case of photolytic processes, these simple kinetics assume that all microorganisms in the population have the same sensitivity to the lethal agent, so when their inactivation is plotted *versus* treatment time, under a constant light irradiation power (I), a straight line would be obtained (pseudo-first order kinetics), but it is known that in real conditions this behaviour can present shoulder and tail deviations (Gyürek and Finch 1998).

Several theories develop alternative kinetics that allow describing non-linear survival curves (Hom 1972; Cerf 1977; Geeraerd et al. 2005). Kinetics curves showing initial shoulder or lag phase deviations indicate that a fraction of surviving microorganisms remains constant in the first instants of treatment, followed by a linear decrease in the number of surviving microorganisms. This is attributed to an inadequate distribution of the UV light through the sample, a delay in the diffusion of the UV light to the bacterial action sites or an initial resistance of the microorganisms to the attack of the disinfectant agent. Tailing-off curves are characterized by an initial rapid linear inactivation phase followed by a slow population decline.

The inactivation kinetics curves that typically show an initial shoulder type deviation (Geeraerd et al. 2000) are adapted to a logarithmic-linear model with one shoulder, indicating that a fraction of surviving microorganisms remains constant in the first instants of treatment, followed by a linear decrease in the number of microorganisms. This model is defined by the equation:

$${N}_{t}=({N}_{o}-{N}_{\mathit{res}})(\left({e}^{-{k}_{max}t}\right).(\frac{{e}^{{k}_{max}S}}{1+{(e}^{{k}_{max}S}-1).{e}^{-{k}_{max}t}})+{N}_{res}$$

where *k*_max_ is the specific inactivation rate constant, *N*_*res*_ is the residual population density, and *S* is the initial stress resistance.

As in the case of the shoulder phenomenon, there are several theories about the occurrence of tails. It may be due to microorganism clusters, to the presence of subpopulations with variable resistance to the disinfectant, either innate or in response to an adaptation to the environment, or also, to a decrease in the concentration of the disinfectant during treatment. There are sigmoidal curves with both linear deviations, showing an initial shoulder phase followed by a linear inactivation phase and ending with a tailing phenomenon.

Mafart et al. (2002) propose the use of Weibull statistical distributions to develop models that include both convex kinetics, with an initial period of no apparent inactivation, and concave kinetics where complete inactivation is not achieved. The Weibull frequency distribution model is based on the distribution of probability designed to describe the behavior of systems that have a certain degree of variability, assuming that microbial populations are heterogeneous in terms of resistance and that each cell requires different conditions to die. Mafart’s model is given by the equation:

$$\mathrm{Log}\frac{{\mathrm N}_{\mathrm t}}{{\mathrm N}_{\mathrm o}}={-(\frac{\mathrm t}{\mathrm\delta})}^{\mathrm P}$$

where δ is the scale parameter and corresponds to the time required to reduce the first logarithmic decimal cycle of the bacterial population and *p* is the shape parameter and indicates the shape of the equation curve, since it takes convex shapes when *p* is greater than 1 and concave when it is less than 1. This model is based on thermal inactivation models and has been used to describe the disinfection on different types of microorganisms in photoreactors, as well as the operational parameters in photolysis (Gomes et al. 2009).

There are two models that describe sigmoidal curves of inactivation; i.e., they describe the behavior of microorganisms when shoulder phenomena, a linear inactivation phase, and tail occur. These models are the biphasic model with shoulder (Geeraerd et al. 2005) and the mixed model of two Weibull-type statistical distributions (Coroller et al. 2006).

The biphasic model with shoulder considers two groups in the microbial population, one having initial stress resistance (shoulder), an initial protection that is gradually destroyed, and a second more resilient population group based on vitalistic or mechanistic models. The vitalistic concept refers to the notion that individuals within a population are not identical and are grouped into populations. This would explain the different UV resistance of microorganisms. The second is a mechanistic concept, which assumes that microorganism inactivation processes are analogous to chemical reactions, which can occur through different pathways (Cerf 1977). The integrated rate equation of the biphasic model is the following:

$$\mathrm{Log}\frac{{\mathrm N}_{\mathrm t}}{{\mathrm N}_{\mathrm o}}=\log(\left(\mathrm f.\mathrm e^{-{\mathrm k}_1\mathrm t}+\left(1-\mathrm f\right).\mathrm e^{-{\mathrm k}_2\mathrm t}\right).\frac{\mathrm e^{-{\mathrm k}_1\mathrm S}}{1+{(\mathrm e}^{-{\mathrm k}_1\mathrm S}-1).\mathrm e^{-{\mathrm k}_1\mathrm t}})$$

where *Nt* represents the bacterial concentration at time t, *No* is the initial concentration of microorganisms (CFU/mL), t is the time, *f* is the fraction of the initial population following the fast reaction, and (1- *f*) is the fraction of the initial population following the second phase of the reaction, where *k*_*1*_ is the rate constant of the sensitive population and *k*_*2*_ is the rate constant of the resistant population. The parameter *S* is the time of the shoulder effect, i.e., the time of the initial stress resistance before bacterial decay.

The mixed model of two Weibull-type statistical distributions proposes that the survival patterns of cells can change with the physiological state of the cells and with how they adapt to stress. It assumes an initially large subpopulation that is more sensitive to stress (first part of the inactivation curve) and a smaller subpopulation that is more resistant to stress (second part of the curve). Its integrated rate equation is:

$${\mathrm{LogN}}_{\mathrm t}=\log(\frac{{\mathrm N}_{\mathrm o}}{1+10^\propto}\left[10^{{-\left(\frac{\mathrm t}{{\mathrm\delta}_1}\right)}^{\mathrm P}+\propto}+10^{{-\left(\frac{\mathrm t}{{\mathrm\delta}_2}\right)}^{\mathrm P}}\right])$$

Like the Weibull model, δ_1_and δ_2_ parameters correspond to the time required to reduce the first logarithmic decimal cycle of the sensitive and resistant bacterial population, *p* indicates the shape of the equation curve, and α is the fraction of the first subpopulation that remains of the total population, defined as α  = log (*f*/(1-*f*)). This model fits sigmoidal curves when δ_2_ tends to infinity and tends to biphasic models when *p* is close to unity and to linear models with tail when δ_2_ tends to infinity and *p* to unity.

### Kinetic modeling of photolytic processes of Clostridium inactivation

**Fig. 5:**
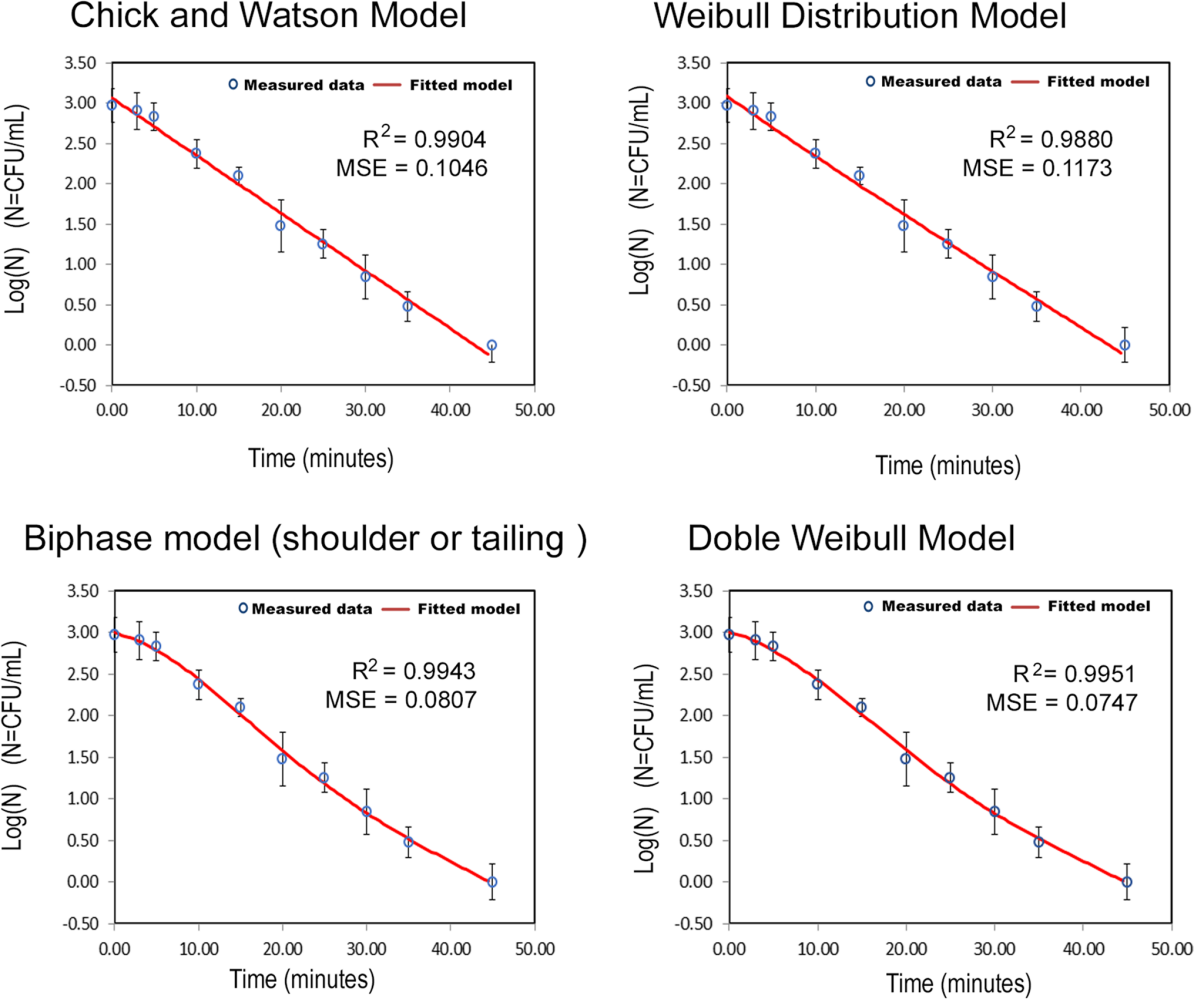
Fits of the integrated rate equations of the models to the experimental kinetic data for *Clostridium* inactivation by photolysis

The inactivation kinetic curves describing the death of *Clostridium* vegetative cells and spores by photolytic treatments of wastewater effluent samples from Monleras macrophyte wetland were analyzed using four mathematical models: (1) the classical Chick-Watson model, (2) the Weibull distribution model, (3) the biphasic model (with tail or shoulder), and (4) the mixed double Weibull model. The classical model of exponential death will tell us if there is a homogeneous population or if there are deviations due to the different resistance of these microorganisms in their different forms. The Weibull distribution model will discriminate the type of curve, either concave or convex, fitting the kinetic data to the corresponding biphasic model curve with shoulder or tail. And finally, the mixed double Weibull model will confirm the type of model that fits the experimental inactivation kinetic curves. The joint count of spores and vegetative *Clostridium* species has been considered for the study of the kinetic mechanism. Figure 5 shows these fits together with the goodness-of-fit parameters for the wastewater samples. Error bars represent the standard deviation of experimental data.

The values of the MSE (close to 0) and *R*^2^ (very close to 1) indicators (Fig. 5), used to measure the goodness of each model fit, show, as a whole, a good fit of two proposed non-linear models (biphasic with shoulder and mixed double Weibull models) to the experimental microorganism inactivation data. However, the linear and Weibull models show an MSE value greater than 0.1, which indicates a worse fit to experimental data by these two models show that there is not a homogeneous population that reacts to stress caused by the photolytic disinfecting action.

The kinetic and statistical parameters for each of the models fitted to the experimental kinetic data for *Clostridium* inactivation by photolysis in wastewater samples are shown in Table 1.

**Table 1:** Kinetic and statistical parameters for the models fitted to the experimental kinetic data for *Clostridium* inactivation by photolysis

Chick-Watson model	*k* (min^−1^)				
	0.16 ± 0.01				
Weibull distribution model	*p*	δ_1_ (min)			
	0.97 ± 0.09	13.5 ± 1.6			
Biphasic model	*k*_*1*_ (min^−1^)	*k*_*2*_ (min^−1^)	*f*		S
	0.24 ± 0.07	0.11 ± 0.05	0.94 ± 0.11		5.3 ± 2.1
Double Weibull model	*p*	δ_1_ (min)	δ_2_ (min)		α
	1.4 ± 0.3	14.5 ± 1.2	31.2 ± 5.7	1.3 ± 0.4	

It is observed that the experimental kinetic curves are not fitted by a linear model but seem to fit a convex curve model, *p* of the mixed double Weibull distribution model are greater than 1, so the curve of *Clostridium* inactivation by photolysis treatment was fitted to a biphasic model with shoulder. The difference of both rate constants (biphasic model) and δ values (mixed double Weibull model) indicates the presence of two *Clostridium* population groups, one resistant to UV radiation with an inactivation rate *k*_*2*_ = 0.11 ± 0.05 min^−1^, the first part of the curve with a shoulder lasting 5.3 ± 2.1 min, and the second one, more sensitive to radiation with a *k*_*1*_ = 0.24 ± 0.07 min^−1^. Thus, the first part of the curve (1-*f* = 6%) shows the UV light irradiation resistant population (*k*_*2*_) during a period that marks the shoulder of *S* = 5.3 min, followed by a rapid inactivation of the sensitive population (*f* = 94%). Finally, the mixed double Weibull model fit confirms a convex curve (*p* = 1.4 ± 0.3) with two populations with a difference between them of α  = 1.3 ± 0.4 log units and with times needed to reduce the first decimal log cycle of the bacterial population of δ_1_ = 14.5 ± 1.2 min and δ_2_ = 31.2 ± 5.7 min, respectively.

The difference in percentages between populations (sensitive and resistance) can be explained by the fact that stress suffered by *Clostridium* vegetative species, an anaerobic species, in the wastewater treatment of the Monleras WWTP, which primary treatment (Imhoff tank) has an anaerobic environment and the macrophyte ponds, with a facultative environment.

As indicated, UV radiation has a high bactericidal capacity on its own, producing severe injury in the cellular genetic material that result in an impediment to DNA replication and the generation of gene mutations, so this mechanism can be explained in vitalistic terms. Thus, initially (shoulder of the curve) radiation doses produce only a few lethal lesions and produce many sublethal lesions that are easily repaired. As increasing the time of the administered radiation dose, a greater number of lethal lesions are produced as a consequence of a greater accumulation of sublethal lesions, leading to a faster rate of cell inactivation. Other factors that could justify this shoulder effect, such as light irradiation power and light distribution in the reactor, are considered to have remained constant over time.

In morphological terms, the spore differs significantly from the vegetative cell, as it is composed in most cases of an outer surface envelope known as exosporium, followed inwards by the protein layers of the envelope and the cortex, which is made up of peptidoglycan, which the vegetative cell lacks (Mitchell 2001). As the cell wall is thicker in spores than in vegetative cells, the disinfection process according to this mechanism must be different for vegetative species (sensitive population) and spores (resistant population), the latter being more resilient. To verify this, samples subjected to photolytic treatment were taken after 100 min, a period longer than that necessary for the reduction of 4 logarithmic cycles (4δ) of microorganisms, which is the classical value considered as a guarantee of food preservation and food safety, hygiene, and quality conditions (Buchanan et al. 1993). After the samples were taken, seeding of the treated water was carried out, increasing the sensitivity of the method. For this purpose, 5 mL of sample was added to 5 mL of culture medium at double concentration instead of 1 to 9 mL of culture medium, and the *Clostridium* vegetative species and spores were identified, observing growth of 9 ± 2 CFU, which indicates that the photolysis process is not effective for the total disinfection of water with presence of *Clostridium*. The presence of the bacteria in the aerobic lagoon opens up new perspectives, since the wild birds may be transporting *Clostridium* from one wetland to another (Long and Tauscher 2006).

### Kinetic modeling of photolytic processes of Coliforms inactivation

**Fig. 6:**
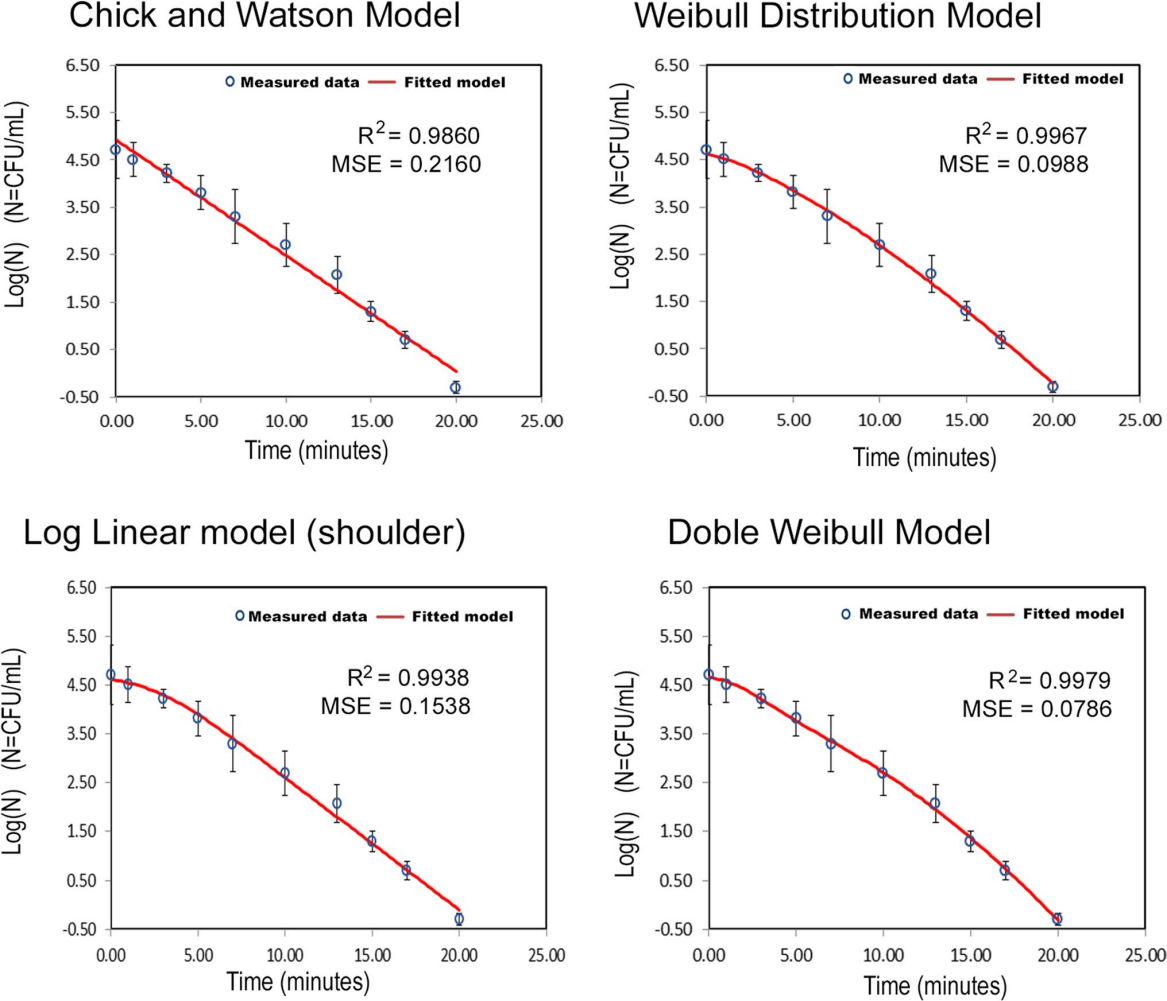
Fits of the integrated rate equations of the models to the experimental kinetic data for total coliforms inactivation by photolysis

**Fig. 7:**
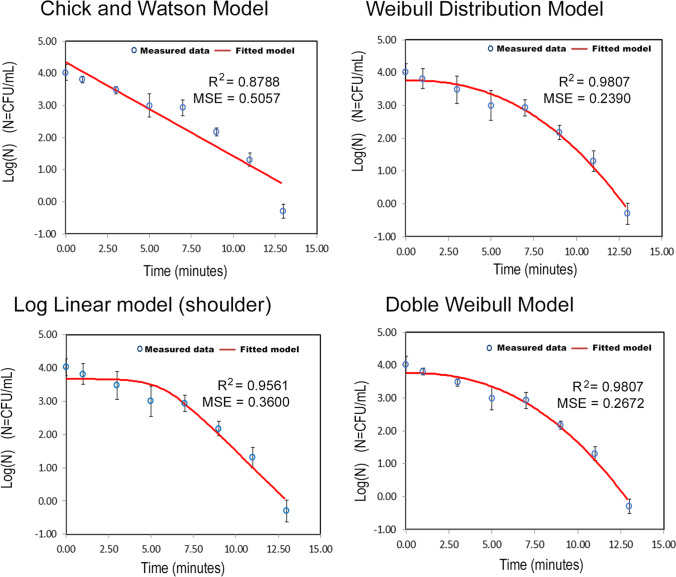
Fits of the integrated rate equations of the models to the experimental kinetic data for Fecal Coliforms inactivation by photolysis

Likewise, the inactivation kinetic curves of coliforms bacteria by photolytic treatments of wastewater samples were analyzed using the different mathematical models indicated (discrimination model). Figure 6 (total coliforms) and Fig. 7 (fecal coliforms) show these fits together with the goodness-of-fit parameters for the wastewater samples from Monleras WWTP.

In short inactivation times, the kinetics follow a log-linear-model; however, when inactivation was followed until times beyond 20 min, it was found that these models demonstrate a better fit to the experimental data obtained, which are reflected in *R*^2^ and MSE values better than those of the linear model (Fig. 6). The biphasic model does not show up in the fits, as despite having a good fit, the equality of the estimated inactivation constants (*k*_1_ and *k*_2_) of the two populations indicates that the biphasic model is unlikely for these data.

The kinetic and statistical parameters for each of the models fitted to the experimental kinetic data for total coliforms inactivation by photolysis in wastewater samples are shown in Table 2.

**Table 2:** Kinetic and statistical parameters for the models fitted to the experimental kinetic data for Total Coliforms inactivation by photolysis

Chick-Watson model	*k* (min^−1^)			
	0.56 ± 0.02			
Weibull distribution model	*p*	δ_1_ (min)		
	1.33 ± 0.07	6.11 ± 0.37		
Log Linear + shoulder model	*k* (min^−1^)	S	4δ (min)	
	0.63 ± 0.03	2.62 ± 0.75	± 17.4	
Double Weibull model	*p*	δ_1_ (min)	δ_2_ (min)	α
	1.7 ± 0.2	4.2 ± 0.9	8.3 ± 1.1	0.43 ± 0.36

On the one hand, statistical fitting confirms that the curve is convex (*p* = 1.7 ± 0.2) with two populations of coliforms with different physiological state. The time required to reduce the first logarithmic decimal cycle of the different types of bacteria (physiological states) are δ_1_ = 4.2 ± 0.9 and δ_2_ = 8.3 ± 1.1 min, respectively. According to the suggested model, total inactivation is reached at 17.4 min.

On the other hand, it is observed that the best kinetic fit is to a logarithmic-linear model with a shoulder. Considering the hypothesis that there are two subgroups with different levels of resistance to stress coexist in a bacterial population, the first part of the curve shows the population resistance to electromagnetic radiation attack during the shoulder period 2.62 ± 0.75 min.

It was verified during the study time that the total elimination of coliforms in the maturation pond is not reached, which indicates that there is photoreactivation by sunlight.

Figure 7 shows these fits together with the goodness-of-fit parameters for fecal coliforms inactivation by photolysis in wastewater samples. Error bars represent the standard deviation of experimental data.

The kinetic and statistical parameters for each of the models fitted to the experimental kinetic data for fecal coliforms inactivation by photolysis in wastewater samples are shown in Table 3.

**Table 3:** Kinetic and statistical parameters for the models fitted to the experimental kinetic data for Fecal Coliforms inactivation by photolysis

Chick-Watson model	*k* (min^−1^)			
	0.67 ± 0.09			
Weibull distribution model	*p*	δ_1_ (min)		
	2.4 ± 0.4	7.3 ± 0.7		
Log Linear + shoulder model	*k* (min^−1^)	S		
	1.15 ± 0.19	5.7 ± 1.0		
Double Weibull model	*p*	δ_1_ (min)	δ_2_ (min)	α
	2.4 ± 0.5	7.3	7.3	4.32

The kinetic study of inactivation of fecal coliform bacteria indicates that, as in the study of total coliform bacteria, it fits a logarithmic-linear model with a shoulder, with a poor fit to the classical Chick-Watson model. It shows an initial electromagnetic radiation resistance (shoulder of the curve) with a duration of 5.7 ± 1.0 min and an inactivation rate of *k* = 1.15 ± 0.19 min^−1^. From a statistical point of view, no difference is observed between the Weibull and double Weibull models, which seems to indicate that fecal coliform bacteria behave as a single population, with 7.3 min being the time needed to reduce the first logarithmic decimal cycle. In the inactivation of fecal coliform bacteria, the shoulder phase observed for a total coliform bacteria concentration (N_o_) with a very smooth decay is attributed to the loss of cell viability following the accumulation of damage during the photolytic process.

### Extrapolation of results from the photolytic reactor to the maturation lagoon

In most of the works presented on the different types of constructed wetlands, emphasis has been placed on the study of the elimination of enteric bacteria such as fecal and total coliforms and *Clostridium perfigens* (Vymazal 2005), either by biological mechanisms such as competition for nutrients and exposure to inhibitory secretions of other bacteria (Stevik et al. 2004), virus-induced lysis (Fischer et al. 2006), and predation by protozoa (Decamp and Warren 1998) or by physical mechanisms, such as filtration and sedimentation as the main physical mechanisms for the removal of pathogenic microorganisms (Alufasi et al. 2017). In this work, we study the influence of chemical mechanisms such as UV solar radiation in real water maturation ponds (without vegetation) in a nature-based wastewater treatment.

In order to compare the results obtained by the photolytic process studied in the reactor and the inactivation processes of aerobic lagoons in the treatment plant, sampling and analysis of coliform and clostridium bacteria were carried out at different points of the treatment plant during the selected months. Samples were collected from the macrophyte wetland and from the tertiary wetland (both inside and at the outlet of the wetland). The average results are shown in Table 4.

**Table 4:** Bacteriological analysis in wastewater treated by nature-based wastewater treatments

	Macrophyte wetland		Maturation lagoon	
	Inside (CFU/100 mL)	Outlet (CFU/100 mL)	Inside (CFU/100 mL)	Outlet (CFU/100 mL)
Vegetative *Clostridia*	950 ± 350	860 ± 230	5 ± 3	1 ± 1
Spore *Clostridia*	35 ± 10	27 ± 8	Not detected	Not detected
Total Coliforms bacteria	52 ± 14. 10^4^	37 ± 17. 10^4^	520 ± 30	490 ± 23
Fecal Coliforms bacteria	16 ± 9. 10^4^	11 ± 9. 10^4^	160 ± 15	120 ± 16

The vegetative species of *Clostridium* and *Coliforms* bacteria were very low in the aerobic lagoon, due to the low vegetation and low water sheet, which favors the germicidal action of sunlight and therefore the elimination of these microorganisms. As can be seen, at the outlet of the maturation lagoon, the total elimination of coliform bacteria is not achieved, which may suggest processes of photo repair or bacterial photoreactivation.

**Fig. 8:**
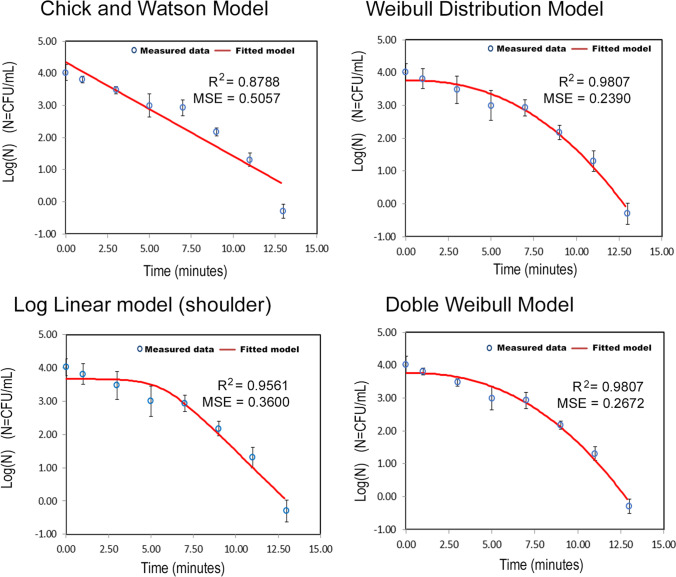
*Coliforms* and *Clostridium* microbial reduction constants

According to the results of the inactivation kinetics obtained, both total coliform bacteria and total sulfite-reducing *Clostridia* can be approximated to the Chick-Watson model. In the case of fecal coliform bacteria, the first 10 min is considered in which the model has a linear behaviour. The population of a species of microorganism exposed to UV light is directly proportional to the intensity I of the radiation and the time t of exposure. The differential rate equation of the Chick-Watson model (Chick 1908):

$$\frac{{\mathrm N}_{\mathrm t}}{{\mathrm N}_{\mathrm o}}=\mathrm e^{-{\mathrm k}_{\mathrm{red}}.\mathrm I.\mathrm t}$$

where *k*_*red*_ is the reduction constant, (cm^2^/mW.s), *I* is the ultraviolet germicidal irradiation (UVGI) (mW/cm^2^), and *t* is the exposure time in seconds. The *k*_*red*_ constant defines the microorganism sensitivity to UVGI and is unique for each species of microorganism under condition studied. The UVGI (Fig. 3) of 51 mW/cm^2^ was considered. Figure 8 shows the determination of the microbial reduction constant for the bacteria studied.

Different reviews have shown that the removal of enteric bacteria occurs in different types of constructed wetlands at a high removal rate 75–99% (Vymazal 2005; López et al. 2019), but the process is extremely slow with first order inactivation rate constants for *E. coli* 0.18 day^−1^ at 22.8 °C (Boutilier et al., 2009), for fecal coliforms 0.177 day^−1^, for total coliforms 0.620 day^−1^, and for *Clostridium* 0.102 day^−1^ (Vymazal 2005). Solar disinfection is usually insignificant in vegetated wetlands due to shading by emergent macrophytes and floating vegetation (Kadlec and Wallace 2009). Recent studies have shown that solar exposure, during warmer seasons and in vegetation-free spaces, decreases by several logarithmic units for *E. coli* contamination in short periods of time (Schmidtlein et al. 2015; Vivant et al. 2016), obtaining values similar to this work in the elimination of enteric microorganisms in summer periods, expressed as a reduction of logarithmic units, thus for fecal coliforms of 2. 0 Ulog in maturation lagoons and 2.5 Ulog in UV systems, for total coliforms of 1.8 Ulog in maturation lagoons and 2.9 Ulog in UV systems and for *Clostridium* of 0.1 Ulog in maturation lagoons and 0.2 Ulog in UV systems (Russo et al. 2019). Furthermore, other authors such as Nguyen et al. (2015) demonstrated that the comparison of vegetated versus pilot-scale open water wetlands seems to provide higher rates of bacterial indicator removal (*E. coli* inactivation rate constant *k* = 2.9 day^−1^ in winter and *k* = 7.0 day^−1^ in summer), mainly through sunlight-mediated inactivation.

The microbicidal action depends on the radiation intensity and the dose applied. The UV dose corresponds to the product of the intensity by the time (mW.s/cm^2^). According to the values obtained for the microbial reduction constant in the photolytic reactor lamp for these species, the bacteriological analysis in wastewater treated by nature-based wastewater treatments (Table 5), and the average radiation intensity during the months of May to July was 31.6 mW/cm^2^, the inactivation time was calculated for maturation lagoon. The inactivation time in photolytic reactor is the time necessary for the reduction of 4 logarithmic cycles (4δ) of microorganisms, while the inactivation time for the maturation lagoon is calculated as a function of the bacterial reduction from the macrophyte wetland outlet and the maturation lagoon outlet, not reaching total water disinfection. Table 5 shows this extrapolation for the maturation lagoon.

**Table 5:** Dose applied for the inactivation of different types of bacteria

	Photolytic reactor		Maturation lagoon	
	Inactivation time (minutes)	Dose applied mW.s/cm^2^	Inactivation time (minutes)	Dose applied mW.s/cm^2^
*Clostridia* bacteria	58	177,480	65.8	124,716
Total Coliforms bacteria	24.4	74,664	18.9	35,799
Fecal Coliforms bacteria	29.2	89,352	20.9	39,756

The efficacy of disinfection (UV dose applied for bacterial load reduction) will depend on several factors including turbidity and organic matter concentration, so the dose applied will depend on the nature of the water (Carré et al. 2018). Authors have reported lower applied dose values than those reported in this paper for coliform bacteria, but these were not extrapolated to total inactivation and used samples that had been filtered or centrifuged prior to UV dosing, such as water from salmonid culture system with an average UV dose of 1821 ± 86 mW s/cm^2^ for a 98% reduction (Sharrer et al. 2005) or swine wastewater lagoons with 700 to 2400 mW s/cm^2^ (Macauley et al. 2006), while in the current work, the samples have not been pre-treated.

The results obtained confirm the photolytic disinfection processes, and the maturation lagoon are effective systems for *Clostridia* bacteria removal after water treatment by nature-based systems. Total removal of coliform bacteria is not achieved by maturation lagoons, but their reduction is significant using low doses of cumulative radiation.

## Conclusions

The inactivation kinetic curves of *Clostridium* bacteria by photolysis fits well in a biphasic model with a shoulder, compatible with two populations, a first resistant one (shoulder of the curve) and a second sensitive one (second part of the curve). This model would be interpreted in terms of a radiation attack on the DNA of the bacterial cell: initially the bacteria would be resistant to radiation, resulting in only a few lethal lesions and many sublethal lesions that would be easily repaired, but as the time of the radiation dose administered is increased, a greater number of lethal lesions would occur, due to more accumulation of sublethal lesions, leading to a faster rate of cell inactivation. It is observed that the *Clostridium* corresponds almost entirely to the sensitive population (94%); this may be due to the fact that the anaerobic and facultative pre-treatment conditions favor the non-formation of spores.

In wastewater after nature-based treatment, the photolytic processes of inactivation of Coliform bacteria are faster than those of *Clostridia* bacteria. Regarding total coliform bacteria, there appear to be two populations with different resistance to stress due to the different physiological states of the bacteria, whereas there is only one population for fecal coliform bacteria.

The extrapolation of the results obtained for the bacterial reduction constants in the photolytic reactor to the maturation lagoons concludes that lower applied UV doses are required to reduce bacterial contamination in these lagoons, being effective systems to reduce bacterial contamination after water treatment by nature-based systems, although complete disinfection is not achieved. Therefore, when implementing this type of low-cost natural treatment for wastewater disinfection, it is the cumulative dose of ultraviolet radiation, and not the treatment time, that should be considered the most important factor. In this sense, with an adequate pre-treatment and maintenance of the plant, low vegetation, and water level in the maturation lagoon, a good performance of disinfection of the wastewater can be obtained, even in the months less favorable for UV radiation due to that during such periods of time a lower organic load enters the maturation lagoons of such nature-based WWTPs, which generally exist in small towns in rural areas, as a consequence of the population seasonality of those municipalities, being necessary a lower radiation dose to reach the bacterial inactivation.

## Data Availability

All data of this study can be obtained from the corresponding author according to appropriate requirements.
